# Biased Recognition of Surprised Facial Expressions Following Awake Craniotomy of a Right Temporal Lobe Tumor

**DOI:** 10.3389/fpsyg.2019.01908

**Published:** 2019-08-20

**Authors:** Akira Midorikawa, Shoko Saito, Chihiro Itoi, Ryuta Ochi, Kentaro Hiromitsu, Ryoji Yamada, Nobusada Shinoura

**Affiliations:** ^1^Department of Psychology, Faculty of Letters, Chuo University, Tokyo, Japan; ^2^Department of Psychology, Graduate School of Letters, Chuo University, Tokyo, Japan; ^3^Institute of Cultural Sciences, Chuo University, Tokyo, Japan; ^4^Department of Neurosurgery, Metropolitan Komagome Hospital, Tokyo, Japan

**Keywords:** perception of surprise, perception of fear, facial expressions, right temporal lobe, awake craniotomy

## Abstract

Lesion studies have shown that the right temporal lobe is crucial for recognition of facial expressions, particularly fear expressions. However, in previous studies, premorbid abilities of the patients were unknown and the effects of epileptic discharge could not be excluded. Herein, we report a case of a patient who underwent assessments of facial recognition before and after brain surgery and exhibited biased recognition of facial expressions. The patient was a 29-year-old right-handed male who underwent an awake craniotomy. Compared with the preoperative assessment, after the surgery, he showed biased recognition of surprised facial expressions, and his ability to recognize other facial expressions either improved or remained unchanged. These findings support the idea that the right temporal lobe is crucial for the recognition of facial expressions of surprise and that functional connectivity between various brain regions plays an important role in the ability to recognize facial expressions.

## Introduction

Functional imaging studies (e.g., Morris et al., 1996) and observations in patients with brain damage (e.g., Adolphs et al., 1994) have provided useful information about the neural basis of the ability to recognize emotional facial expressions. The findings of these studies suggest that the perception of facial expressions is a fundamental role of the temporal lobe in humans, particularly the bilateral amygdala region. For example, a classic study found that the right hemisphere was crucial for the recognition of facial expressions (Bowers et al., 1985); however, the authors did not find any apparent differences in the types of emotion or provide information about brain regions other than designating the right hemisphere. Subsequent human lesion studies found that the perception of fear was lateralized to the right hemisphere (Adolphs et al., 2001; Meletti et al., 2003), suggesting that the right temporal lobe, particularly the amygdala, is crucial for the recognition of fear. However, previous lesion studies have several limitations. First, many included patients with epilepsy related to congenital disorders. Epileptic discharges may alter nervous system function in peripheral brain regions, so patients with congenital disorders may have abnormal developmental trajectories. Indeed, without surgery, some patients with temporal lobe epilepsy have demonstrated deficits in emotion recognition (Meletti et al., 2009). Furthermore, Tanaka et al. (2013) found no differences in emotional recognition tasks involving the face between epileptic patients who did and those who did not undergo lobectomy. Taken together, these findings suggest that epileptic discharges may play a crucial role in disorders involving the recognition of emotional facial expressions. Second, most surgical case studies did not measure or report preoperative ability, and relatively few studies have evaluated preoperative ability in patients with temporal lobe epilepsy (Yamada et al., 2005; Shaw et al., 2007; Amlerova et al., 2014). Yamada et al. (2005) and Shaw et al. (2007) observed deficits in the ability to recognize facial expressions in patients with temporal lobe epilepsy during the preoperative period. Amlerova et al. (2014) reported that temporal lobe surgery had no effect on patients’ ability to recognize emotion based on facial expressions. Taken together, these findings suggest that temporal discharges, rather than the temporal lobectomy, may be the critical factor that alters one’s ability to recognize emotion conveyed by facial expression. Therefore, the present study assessed this ability in a non-epileptic patient with no history of congenital disorders who presented with a temporal lobe tumor and exhibited discrepancies in his ability to recognize emotions based on facial expressions between his pre- and post-operative assessments. In addition, this article discusses the role that the lateralization of the medial temporal lobe plays in this deficiency.

## Materials and Methods

### Subject

**FIGURE 1:**
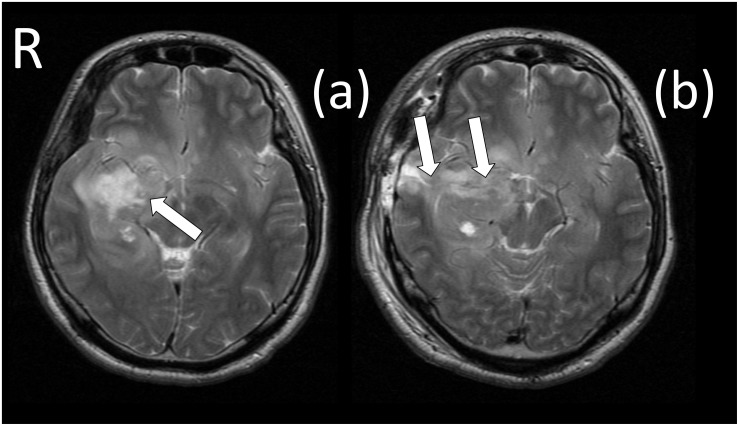
Magnetic resonance imaging (MRI) scans before and after surgery. **(a)** The white arrow identifies the brain tumor (meningioma), which was located in the right temporal region. **(b)** The white arrows identify the surgically induced scars that reached to the amygdala.

The patient was a 29-year-old, right-handed male. Following graduation from graduate school, he worked as an office worker for 5 years. After experiencing olfactory hallucinations, he visited a local hospital and was diagnosed with a suspected brain tumor. To diagnose and treat the tumor, he was hospitalized at Tokyo Metropolitan Komagome Hospital. Magnetic resonance imaging (MRI) showed a right temporal lobe lesion (see Figure 1a). Neuropsychological examination showed several cognitive impairments (see Table 1). The patient showed deterioration in visual memory relative to verbal memory and a severe recall deficit on the Japanese version of the revised Wechsler Memory Scale (WMS-R). However, based on the Raven’s Colored Progressive Matrices (RCPM) and the Rey–Osterrieth Complex Figure Test (ROCFT), the patient demonstrated well-preserved intelligence, visuospatial function, and everyday memory. In addition to the routine tests, we administered an in-depth test of emotional functioning. In December 2009, an awake brain surgery was performed to diagnose and remove the lesion and the extent of the surgical procedure reached the amygdala (see Figure 1b). The final diagnosis was a meningioma. Written informed consent was obtained from the patient for the publication of this case report.

**TABLE 1:** Neuropsychological profile of the patient.

	**Before surgery**	**After surgery**	**Max**
**Raven’s Colored Progressive Matrices (RCPM)**	35	36	/36
**Mini-Mental State Examination (MMSE)**	30	27	/30
**Standard Language Test of Aphasia (SLTA)**			
Naming	20	20	/20
Repetition	5	5	/5
Comprehension	10	10	/10
**Wechsler Memory Scale-revised (WMS-R)**			
Verbal memory	106	125	
Visual memory	91	116	
General memory	105	126	
Attention and concentration	133	127	
Delayed recall	59	112	
**Ray–Osterrieth Complex Figure Test (ROCFT)**			
Copy	36	36	/36
Immediate recall	32	31	/36
**Digit Span**			
Forward	7	6	
Backward	7	5	
**Face identification**	12	13	/14

### Recognition of Facial Expression (Pre- Post-examination)

The patient completed a recognition test of emotional facial expressions before and after surgery to examine his abilities in this regard. The pre-operative session was performed 3 days prior to the surgery and the post-operative session was performed 4 days after the surgery; therefore, the period between the pre- and post-operative assessments was 7 days. For reference, control subjects were also administered the facial recognition test. The control subjects were recruited from graduate and undergraduate courses and consisted of 23 healthy male volunteers (mean age: 28.6 ± 2.10 years) with no history of neurological disorder. Before the examination, all of the control subjects were informed of the aims of the test, and the authors obtained their written informed consent. The tests were administered at two timepoints with an interval of 1 week, which corresponded with the patient’s interval between tests. The facial expression stimuli included six emotion groups and a neutral group, yielding a total of 54 pictures. The presented emotions and the corresponding numbers of pictures for each emotion were digitalized from the pictures used by Ekman and Friesen (1975) as follows: happiness (10 pictures), sadness (9 pictures), surprise (8 pictures), fear (8 pictures), disgust (8 pictures), anger (8 pictures), and neutrality (3 pictures). Each of the pictures appeared in grayscale [262 pixels (height) × 184 pixels (width)] and was presented individually on a 12.1-inch monitor of a laptop computer (VAIO Type G, Sony Corporation; Tokyo, Japan). The subject was asked to choose the type of emotional expression from a target sheet that contained a list of six possible emotional words and the word “neutrality.” During the sessions, the target sheet was placed on the desk next to the computer which was located approximately 50 cm from the subject but the head of the subject was not fixed. Each of the stimuli was displayed until the subject selected an emotion by responding either verbally or by pointing to the sheet. As an indication of the effects of surgery on the recognition of facial expressions, we used subtraction scores (the pre-examination scores were subtracted from the post-examination scores).

### Statistical Analyses

Modified *t*-tests were used to compare our case with control subjects (Crawford and Howell, 1998; Crawford and Garthwaite, 2005). Modified *t*-tests are optimized for use in single-case studies, and the algorithm is widely used in neuropsychology (e.g., Krämer et al., 2013).

### Ethics Statement

This study was approved by the Ethics Committee of Tokyo Metropolitan Komagome Hospital (reference number: 1736).

## Results

**FIGURE 2:**
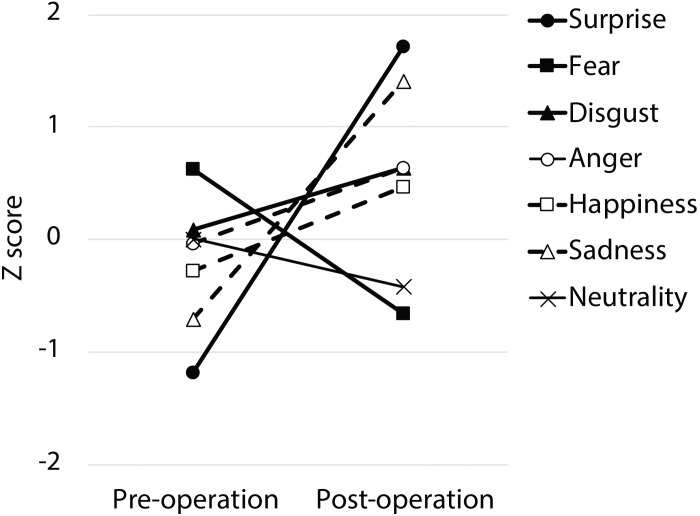
The pre- and post-operative z-scores. The partial credit score for each facial expression was converted into a z-score for each time period based on normative data obtained from 21 normal control subjects.

**FIGURE 3:**
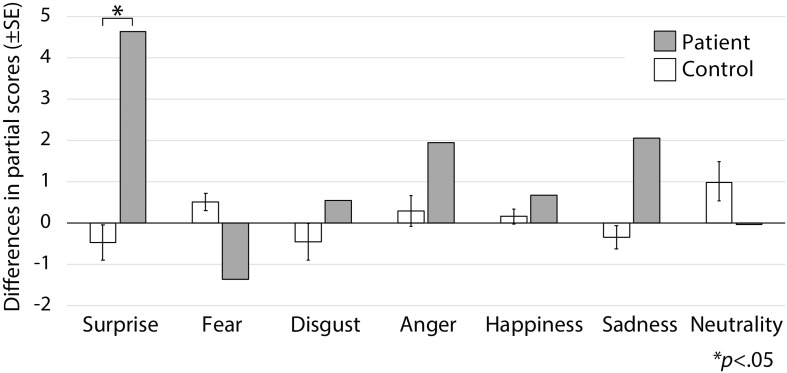
Subtraction score and modified *t*-tests. Modified *t*-tests revealed that the patient’s subtraction score for surprise was significantly larger than that of the control subjects, which indicates that his score improved after surgery.

Following surgery, the patient did not display any obvious impairments in standard cognitive abilities, including memory and verbal functions (see Table 1), but an assessment of his ability to recognize emotional expressions revealed differences compared with his performance before surgery (Figure 2). Due to missing values in two control subjects, the statistical analyses were performed using 21 of 23 control subjects. In addition, during the facial expression recognition task, several of the control subjects responded using different names for the stimuli other than the original names provided by Ekman and Friesen (1975) (Tables 2, 3). Thus, to maximize the intrinsic value of the stimuli, a “partial credit score” (Heberlein et al., 2004; Atkinson et al., 2007) was adopted for later analyses (see Supplementary Tables S1–S3). In the scoring method, each response was given credit based on the proportion of subjects in the reference group giving that response. For example, if a given stimulus was called “happy” by 50% of the reference group, “angry” by 40%, and “neutral” by 10%, then the response “happy” would receive a score of 1.0 (0.5/0.5), “angry” would receive 0.8 (0.4/0.5), and “neutral” would receive 0.2 (0.1/0.5). All other answers would receive 0 (Heberlein et al., 2004). Subsequently, modified *t*-tests revealed that the patient’s subtraction score for surprise was significantly larger than that of the control subjects [*t*(20) = 2.56, *p* = 0.019, two-tailed], which indicates that the score improved after surgery. On the other hand there were no significant differences for the other stimuli [sadness: *t*(20) = 1.82, *p* = 0.085, two-tailed; fear: *t*(20) = −1.90, *p* = 0.071, two-tailed; disgust: *t*(20) = 0.484, *p* = 0.63, two-tailed; anger: *t*(20) = 0.941, *p* = 0.37, two-tailed; happiness: *t*(20) = 0.593, *p* = 0.56, two-tailed; and neutrality: *t*(20) = −0.495, *p* = 0.63, two-tailed]. Taken together, these results indicate that the patient’s recognition of the facial expressions of surprise improved after surgery beyond a learning effect (see Figure 3).

**TABLE 2:** Percentage score matrix of the expected and observed responses to facial expressions in the control subjects.

		**Responded to**						
		**Surprise**	**Fear**	**Disgust**	**Anger**	**Happiness**	**Sadness**	**Neutrality**
Expected to	Surprise	91.7	2.4	0.0	0.0	1.2	0.6	4.2
		92.9	1.8	0.0	0.6	0.6	0.0	4.2
	Fear	55.4	25.6	4.8	8.3	0.0	5.4	0.6
		51.8	27.4	7.7	9.5	0.0	3.6	0.0
	Disgust	0.6	0.6	45.2	37.5	1.8	0.6	13.7
		0.0	3.0	36.9	42.3	1.2	0.0	16.7
	Anger	4.2	2.4	13.7	76.2	0.0	0.0	3.6
		3.0	1.2	14.3	75.0	0.0	0.0	6.5
	Happiness	20.0	0.5	3.3	1.4	67.1	0.0	7.6
		18.6	1.4	3.3	0.0	68.6	0.0	8.1
	Sadness	0.5	4.2	17.5	1.1	0.0	71.4	5.3
		0.5	5.3	18.0	1.6	0.5	69.3	4.8
	Neutrality	3.2	1.6	4.8	15.9	0.0	0.0	74.6
		1.6	0.0	7.9	9.5	1.6	0.0	79.4

*Top values, first trial; bottom values, second trial performed after a 1-week interval. Gray color means the observed responses were matched with the expected targets.*

**TABLE 3:** Percentage score matrix of the expected and observed responses to facial expressions in the patient.

		**Responded to**						
		**Surprise**	**Fear**	**Disgust**	**Anger**	**Happiness**	**Sadness**	**Neutrality**
Expected to	Surprise	75.0	12.5	0.0	0.0	0.0	0.0	12.5
		100.0	0.0	0.0	0.0	0.0	0.0	0.0
	Fear	50.0	37.5	0.0	0.0	0.0	12.5	0.0
		87.5	12.5	0.0	0.0	0.0	0.0	0.0
	Disgust	0.0	0.0	50.0	37.5	0.0	0.0	12.5
		0.0	0.0	50.0	37.5	0.0	0.0	12.5
	Anger	12.5	0.0	12.5	75.0	0.0	0.0	0.0
		0.0	0.0	0.0	100.0	0.0	0.0	0.0
	Happiness	10.0	0.0	0.0	0.0	70.0	0.0	20.0
		20.0	0.0	10.0	0.0	70.0	0.0	0.0
	Sadness	0.0	0.0	33.3	0.0	0.0	55.6	11.1
		0.0	0.0	11.1	0.0	0.0	88.9	0.0
	Neutrality	0.0	0.0	0.0	33.3	0.0	0.0	66.7
		0.0	0.0	0.0	0.0	0.0	0.0	100.0

*Top values, pre-surgery; bottom values, post-surgery. Gray color means the observed responses were matched with the expected targets.*

**FIGURE 4:**
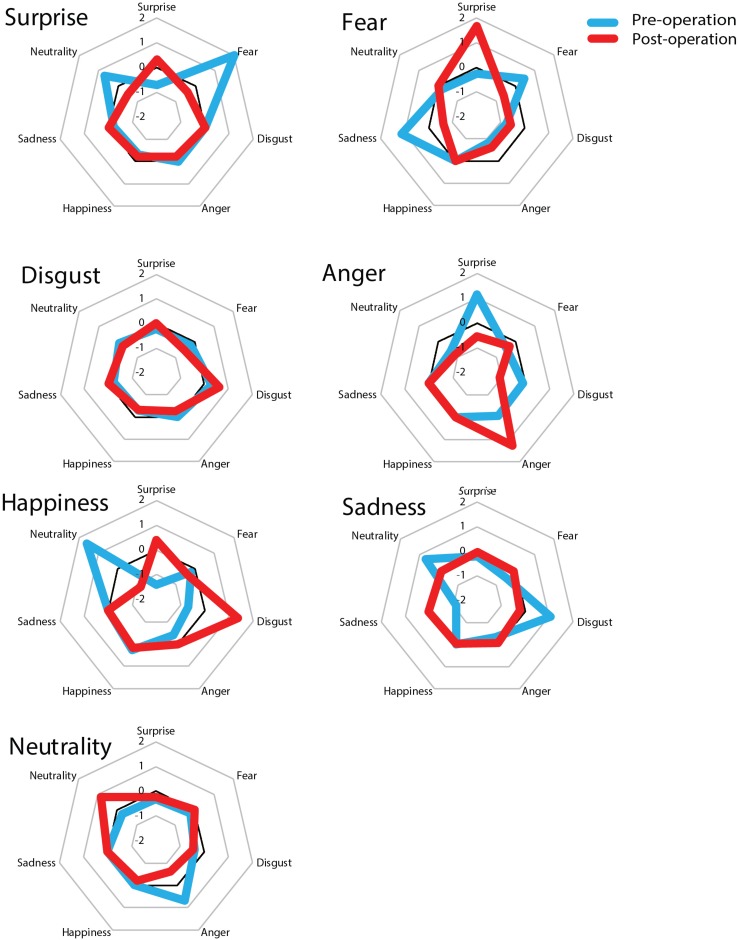
z-scores of the partial credit scores before and after surgery. The large title refers to the original name of the stimuli and the small title of each of the seven squares represents the responses of patients and controls. The score was converted into a z-score based on 21 normal control subjects. Before surgery, the patient showed a fear bias for surprise stimuli whereas after surgery he showed a surprise bias for fear stimuli. The other six facial expressions remained unchanged compared to before surgery.

Furthermore, to evaluate the accuracy of the patient’s performances between pre- and post-surgery, a z-score of the partial credit score was calculated based on the average of two time points in control subjects (see Figure 4). The z-score indicated that prior to surgery the patient responded to the surprise target as would be expected to a fear target (*z* = 2.01, *p* = 0.04, two-tailed) whereas after surgery his score was within 1 SD (*z* = −0.32, *p* = 0.75, two-tailed). On the other hand, after surgery the patient responded to fear stimuli as would be expected to a surprise target much more than control subjects; however, the difference was not statistically significant (pre: *z* = −0.26, *p* = 0.79; post: *z* = 1.67, *p* = 0.09, two-tailed). These results imply that his facial recognition ability was impaired in that his recognition of surprised facial expressions was biased. We found no significant differences for other facial expressions (*p* > 0.10).

## Discussion

We examined the role of the right temporal lobe in the recognition of facial expressions before and after an awake craniotomy. Following the surgery, it was confirmed that the extent of the operation affected the amygdala of the patient and that he showed biased recognition of surprised facial expressions. Compared to previous research, this study has a number of advantages. First, because the patient’s preoperative status was known, we were able to compare the recognition ability before and after surgery. Second, the surgical procedure used was awake craniotomy. Compared to surgery under general anesthesia, awake craniotomy for brain tumors improves safety in terms of preserving neurological function (Sacko et al., 2011). In addition to brain mapping, the use of continuous monitoring of neurological function during awake surgery appears to be important because, based on the brain mapping of negative lesions, it is possible that neurological function deteriorates during tumor resection. Therefore, we continuously monitored neurological functions, including the ability to recognize the facial expressions of emotions. Without this additional monitoring, no apparent neuropsychological deficit would have been observed following the surgery. We therefore believe that our results were not due to secondary effects of a neurological deficit but reflect a primary effect of the surgical lesion. Previous studies have shown that the right hemisphere, particularly the amygdala region, plays an important role in the recognition of facial expressions, including fear (Adolphs et al., 2001; Meletti et al., 2003). Although previous case studies have several limitations, given our findings, it is possible to conclude that a right amygdala lesion will contribute to biased recognition of surprised facial expressions.

It is easy to confuse a surprised facial expression with a fearful one and this phenomenon is related to activity in the right amygdala region (Kim et al., 2003). As shown in Figure 4, it is possible that before surgery the present patient had some confusion regarding the recognition of a surprised expression, and as a result, he responded much more to fear instead of surprise relative to control subjects. On the other hand, after surgery, his recognition of surprise increased and was accurate as his score was equivalent to that of controls. However, he also showed an exaggerated response to fearful expressions, which may have been due to the emergence of a fear bias following resection of the right temporal lobe tumor, which included the amygdala region; consequently, he was biased in response to surprised facial expressions. However, it is not possible to attribute this change to the direct effects of his primary lesion in the right temporal lobe (including the amygdala) and it is likely that multiple regions are important for understanding the present results.

The present findings are similar to those of Shaw et al. (2007), who examined a variety of social abilities in patients who had undergone an anterior temporal lobectomy including the amygdala region. The authors reported that left resection improved the ability to recognize fear, disgust, and sadness but deteriorated the ability to recognize happiness, anger, and surprise. On the other hand, right resection improved the ability to recognize sadness, happiness, and disgust but deteriorated the ability to recognize fear and surprise. With respect to fearful and sad expressions, left anterior temporal lobectomy improved the ability to recognize fear but reduced the ability to recognize sadness while a right lobectomy resulted in a slight decline in fear recognition but a small improvement in the recognition of sadness. Thus, the findings of Shaw et al. (2007) imply that, as in the present case, a temporal lobectomy may result in deficits as well as improvements in the recognition of emotion, even after a cortical resection.

The right amygdala, right posterior central gyrus, and left insula are crucial for the recognition of surprised facial expressions (Zhao et al., 2017). Changes after surgery may be explained by the release-of-function phenomenon (Martin et al., 2000), which postulates distal metabolic and neurophysiological normalization following the excision of an epileptogenic focus (Shaw et al., 2007) or by paradoxical functional facilitation (PFF), a similar notion proposed by Kapur (1996), which attributes improved performance to a reduction in inhibitory signals triggered by damage to specific brain regions. Based on these concepts, it is reasonable to hypothesize that the changes in our patient’s ability to recognize surprise after surgery was due to a reduction in imbalances in the brain network.

Alternatively, several other factors may explain the changes observed in the present study. At his preoperative assessment, the patient exhibited deterioration in the ability to recognize facial expression of surprise. Imaging studies of the amygdala region have revealed activation during the identification of not only fearful facial expressions but also surprised facial expressions (Kim et al., 2003). Therefore, the present patient may have experienced functional deterioration even before his surgery. Furthermore, after his surgery, the patient characteristically exhibited an overestimation of surprised facial expressions as fear (see Figure 4). This may have been due to a low sensitivity for fear as well as hypersensitivity to surprise. Normal populations tend to confuse fear with surprise (Table 2), but not with other emotions, and it has been suggested that this phenomenon is based on perceptual-attentional limitations that may be due to an impaired ability to identify or pay attention to the relevant cues that would aid in the recognition of fearful and surprised facial expressions (Roy-Charland et al., 2014). Thus, after his surgery, the present patient may have experienced an attentional shift with respect to the cues necessary for the recognition of facial expressions. The present findings imply that damage to the right temporal lobe that includes the amygdala region following the resection of a tumor can induce slight disturbances in the ability to recognize fearful facial expressions and biases in the recognition of other facial expressions, particularly surprise.

There are several limitations to the present study that must be noted. First, the present patient was diagnosed with a brain tumor in the temporal region that may have influenced his cognitive abilities. Prior to surgery, the patient exhibited poor recognition of surprised facial expressions as well as poor performance on a delayed recall task. The right amygdala region is related to the recognition of surprised faces (Kim et al., 2003), and thus it is plausible that the present patient had functional disturbances in the right temporal regions prior to his surgery. However, he showed improvements in both functions following surgery and it is possible that the effects of his tumor on the recognition of fear were limited. Second, the present study was based on a single case, and our results were based on a small number of changes. Because different people vary in their ability to recognize facial expressions (e.g., Isaacowitz et al., 2007), definitive conclusions regarding the neural bases underlying the recognition of facial expressions cannot be made based on the results of a single study. Third, the procedure used in the present study had some covert problems, including the fact that the test–retest interval of the patient was 7 days and a learning effect may have influenced the results. However, our patient did not receive feedback regarding his answers; therefore, any learning effect had a limited influence on the results. Furthermore, we adopted a scoring method that corresponds to a partial credit score (Heberlein et al., 2004; Atkinson et al., 2007) allowing for the easy detection of variability among the responses of the control group, particularly when scores were low. In fact, the score of the control group for fearful expressions was relatively low (see Table 2), which suggests that there may have been a floor effect. Recent research suggests that Ekman’s six basic emotional expressions are not universal phenomena, including in Japanese people (Sato et al., 2019). This finding may affect lesion studies, including the present study, and therefore it is necessary to consider cultural differences in future studies.

In conclusion, the present study identified discrepancies in the ability of the patient to recognize facial expressions before versus after the awake craniotomy. Compared to before the surgery, the patient exhibited changes in the ability to recognize surprise. It is our opinion that these results were not due to changes in independent regional function but to disturbances in functional connectivity between the damaged right temporal lobe and the preserved left temporal lobe and among other regions.

## Data Availability

The datasets generated for this study are available on request to the corresponding author.

## Ethics Statement

This study was approved by the Ethics Committee of Tokyo Metropolitan Komagome Hospital (reference number: 1736).

## Author Contributions

AM designed the study, conducted the research, analyzed the data, and wrote the manuscript. SS and CI contributed to the research in their clinical capacity and helped with manuscript preparation. KH contributed to the research on normal subjects. RO contributed to the data analyses and manuscript preparation. RY and NS contributed to the manuscript preparation.

## Conflict of Interest Statement

The authors declare that the research was conducted in the absence of any commercial or financial relationships that could be construed as a potential conflict of interest.
